# Non-Invasive Hybrid Ultrasound Stimulation of Visual Cortex In Vivo

**DOI:** 10.3390/bioengineering10050577

**Published:** 2023-05-10

**Authors:** Chen Gong, Runze Li, Gengxi Lu, Jie Ji, Yushun Zeng, Jiawen Chen, Chifeng Chang, Junhang Zhang, Lily Xia, Deepthi S. Rajendran Nair, Biju B. Thomas, Brian J. Song, Mark S. Humayun, Qifa Zhou

**Affiliations:** 1 Department of Biomedical Engineering, University of Southern California, Los Angeles, CA 90089, USA; cgong841@usc.edu (C.G.); runzeli@usc.edu (R.L.); gengxilu@usc.edu (G.L.); jji38749@usc.edu (J.J.);; 2 USC Roski Eye Institute, Keck School of Medicine of University of Southern California, Los Angeles, CA 90033, USA; 3 Department of Neurobiology, University of Southern California, Los Angeles, CA 90089, USA

**Keywords:** vision restoration, optic nerve damage, non-invasive, ultrasound stimulation, visual cortex, electrophysiology, photometry

## Abstract

The optic nerve is the second cranial nerve (CN II) that connects and transmits visual information between the retina and the brain. Severe damage to the optic nerve often leads to distorted vision, vision loss, and even blindness. Such damage can be caused by various types of degenerative diseases, such as glaucoma and traumatic optic neuropathy, and result in an impaired visual pathway. To date, researchers have not found a viable therapeutic method to restore the impaired visual pathway; however, in this paper, a newly synthesized model is proposed to bypass the damaged portion of the visual pathway and set up a direct connection between a stimulated visual input and the visual cortex (VC) using Low-frequency Ring-transducer Ultrasound Stimulation (LRUS). In this study, by utilizing and integrating various advanced ultrasonic and neurological technologies, the following advantages are achieved by the proposed LRUS model: 1. This is a non-invasive procedure that uses enhanced sound field intensity to overcome the loss of ultrasound signal due to the blockage of the skull. 2. The simulated visual signal generated by LRUS in the visual-cortex-elicited neuronal response in the visual cortex is comparable to light stimulation of the retina. The result was confirmed by a combination of real-time electrophysiology and fiber photometry. 3. VC showed a faster response rate under LRUS than light stimulation through the retina. These results suggest a potential non-invasive therapeutic method for restoring vision in optic-nerve-impaired patients using ultrasound stimulation (US).

## 1. Introduction

Retinal abnormalities that cause severe visual impairment (retinal dystrophy, degeneration, retinal detachment, etc.) are confined to their primary cellular structures and cause the impairment of other structures, including the visual pathway and visual cortex in the brain [1,2]. Common optic nerve disorders include glaucoma, optic neuritis, and ischemic optic neuropathy [3,4,5,6,7,8,9]. Glaucomatous optic neuropathy is the world’s second-most common blinding eye disease after cataracts. In addition, other situations that adversely affect the optic nerve, such as inflammation, vascular abnormalities, malignancy (cancer), and trauma, can also cause permanent optic nerve damage and vision loss. There have been some attempts to restore vision with retinal prostheses, such as the Argus II [10,11] and US-induced electrostimulation prostheses [12]. These methods rely on electrode matrices to deliver cluster electrical stimulation and, as technology has evolved, have even become wireless, decoupling the stimulator from the host [13,14,15,16].

Due to limitations in the size of the retina and electrode spacing of the retinal prosthesis, this technology is limited by the resulting low resolution. Additionally, difficulties with biocompatibility and the effect of immune rejection also limit the potential impact of retinal prostheses. Furthermore, these prostheses rely on the normal function of the optic nerve pathway. Therefore, those patients with optic nerve dysfunction will not be able to benefit from a retinal prosthesis.

These barriers have led researchers to look for other methods to rehabilitate the visual system. Historically, one of the first places to be considered for a visual prosthetic was the VC because of its potential to act as an interventional site for virtually all forms of blindness, including those due to optic nerve trauma and damage to the pre-cortical areas. The first studies by the German ophthalmologist Foerster [17] in the 1930s confirmed that the direct electrical stimulation of the VC allowed a completely blind person to perceive some points of light. Notably, phosphorescence from a fixed point on the cerebral cortex is localized to a corresponding point in the visual field, and this could be evoked even in blind patients. With a single electrode, it evokes a tiny white spot at a fixed point in the visual field. Similarly, with multiple electrodes, two or more such spots could be evoked.

With such attempts, patients can partially restore their vision by stimulating the visual cortex. There are now many ways to stimulate the cortex. Electrical stimulation and optogenetic stimulation offer good spatial resolution and stimulation precision, but they typically require invasive surgeries to insert electrodes or fibers into the brain [18,19], carrying potential risks of infection and other complications. Transcranial magnetic stimulation (TMS) is a non-invasive stimulation method, but it often lacks good precision for stimulating specific brain regions.

Transcranial focused ultrasound (tFUS) has emerged as a potentially effective method for cortical stimulation. It possesses a unique ability to target highly specific brain regions with considerable spatial resolution, all without the necessity of inserting wires or electrodes into the brain. The use of ultrasound phased arrays can help overcome the challenges posed by the skull in tFUS by calculating the phase compensation needed for the ultrasound phased array in tFUS through theoretical modeling and numerical simulation to achieve better transcranial focusing effects. Non-invasive tFUS technology that does not require a craniotomy is becoming increasingly feasible, and a non-invasive US-based VC prosthesis would help eliminate most of the complications of electrical-based VC prostheses [20,21,22,23,24,25,26,27].

Li’s [28] work generated 15 MHz ultrasound waves based on the photoacoustic effect for VC stimulation with a stimulation accuracy of 85 µm, but expanding it to a large array for multi-point neural modulation is limited by the complex control of optical instruments, making it difficult to reduce the device’s size in the short term. The development of flexible PZT arrays will make it possible to provide multi-point ultrasound stimulation. Here, we attempted to characterize the effects of non-invasive, low-intensity, low-frequency US and focused on neuromodulation in rats by using a PZT ring ultrasound transducer to stimulate the VC. The advantage of this type of US is that it does not require a craniotomy or skull thinning, which is usually necessary for other US methods to be effective in neuromodulation without attenuating the response. By comparing the neuronal changes evoked by direct visual cortex ultrasound stimulation to those evoked by optical stimulation of the eye, it could be concluded that ultrasound is able to evoke neuronal responses without damaging the skull. Although the current biomolecular and cellular mechanisms underlying the focused ultrasound (fUS)-excited mammalian neuronal response remain unclear, previous studies have shown that some specific calcium-selective mechanosensitive ion channels are sensitive to ultrasound. In contrast to transcranial magnetic stimulation (TMS) and transcranial direct current stimulation (tDCS) [29,30], fUS can be adjusted in real time based on feedback from the transducer and penetrance to achieve a greater stimulation precision and depth [31]. In addition, fUS is chemically and genetically free of harmful side effects on the organism [32,33]. Therefore, non-invasive US would provide a potential non-surgical way to restore vision in blind patients with optic nerve damage, which is a better alternative to existing electrical stimulation prostheses.

## 2. Materials and Methods

### 2.1. Animal Preparation

All animal procedures were approved by the University of Southern California Institutional Animal Care and Use Committee (IACUC 21084). Three-month-old male and female Long-Evans (LE) rats (*n* = 10) were used for this study. The rats were initially anesthetized with an intraperitoneal injection of Ketamine/Xylazine (50–90 and 5–10 mg/kg) and then with sevoflurane inhalation through a nose cone [34]. The eyes were dilated using 1% tropicamide and 2.5% phenylephrine drops. The cranium was exposed by removing the skin above the skull, and a small cranial hole was made using a dental drill to expose the visual cortex (VC). All procedures and experiments were performed in a dark room illuminated with dim red light to minimize possible stimulation of the VC due to photoreceptor activation. The space between the brain surface and transducer was filled using ultrasound gel.

### 2.2. Transducer Design and Acoustic Field with Skull

A 3.6 MHz ring transducer with a focal depth of 23 mm was used for this experiment. Ultrasound penetration is stronger at lower frequencies than at higher frequencies [25,26]. A collimator with a depth of 20 mm was used to ensure that the positioning of the ultrasound focus was exactly on the visual cortex. The sound field distribution of a ring transducer was simulated on the Field II (version 3.30) computing platform and MATLAB (R2020a, Mathworks, Natick, MA, USA) software. Results were generated using an axisymmetric model with circular symmetry.

**Figure 1:**
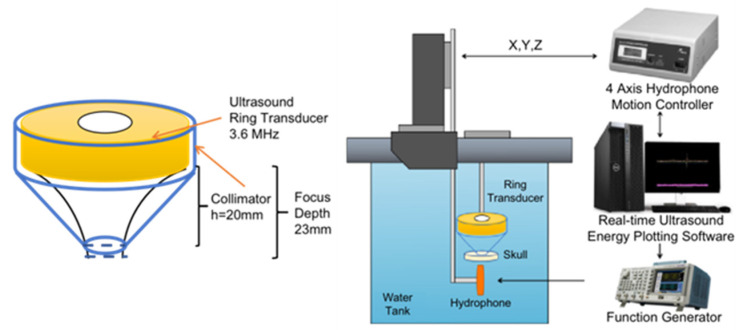
A figure of the 3.6 MHz ring transducer with the collimator and the hydrophone system setup.

To measure the ultrasonic sound intensity and map the field, the source’s axial and transverse profiles were measured under linear propagation conditions using a needle hydrophone (HGL-0400, ONDA, Sunnyvale, CA, USA). The needle hydrophone tested the spatial distribution of acoustic intensity to obtain these measurements. To measure the transducer’s spatial location, a collimator was securely fixed to the transducer, and the positioning system rod was attached to the hydrophone. A linear scanning ultrasound bio-microscopy (UBM) system was used for acoustic field measurements (Ext clock 10 MHz, sampling rate 1800 MHz, Speed Bmode). The FUS input parameters were 3.6 MHz sine wave in Burst mode, 250 ms Burst cycle, and about 15.8 V amplitude (50 mV signal generator with a 50 dB signal amplifier). In order to measure the real distribution of the acoustic intensity with the block of the skull, a real rat skull was attached to the collimator, and then the hydrophone was tested again. Figure 1 shows the layout of the devices. The stimulation results of the ring transducer were also analyzed in MATLAB.

### 2.3. The System Setup of Fiber Photometry Signal Acquisition and Electrophysiological Recordings

#### 2.3.1. Fiber Photometry Signal Acquisition

The activity of neurons is closely related to their internal calcium ion concentration, and neurons burst with a brief peak of calcium ion concentration during firing. Fiber-optic calcium imaging systems, such as the one used in this study, are highly specific and can be utilized to characterize the visual neuronal responses in the visual cortex region without being confused by responses in other brain regions, such as the auditory regions. These systems have higher specificity and resistance to noise interference than electrophysiological systems [35]. The combination of two systems, namely, fiber-optic calcium imaging and electrophysiological acquisition, ensures both the accuracy and noise immunity of the signal.

For calcium, a 2 mm diameter hole was made above the rat’s primary VC, and a microinjector (RWD R-480 microinjector) was used to inject 0.5 uL of the GCAMP8 (pGP-AAV-syn-jGCaMP8m-WPRE, titer ≥ 1 × 10^13^ vg/mL) virus into the primary visual cortex. The injection time was 30 min, and then the rat was allowed to recover for 30 days.

**Figure 2:**
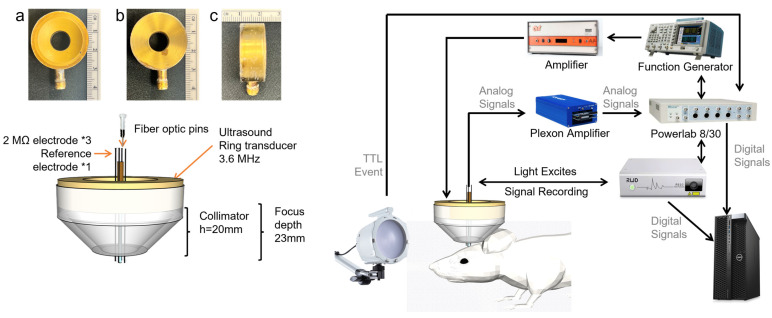
The system setup of electrophysiological signal and fiber. (**a**) The frontal aspect of the ring transducer; (**b**) the posterior aspect of the ring transducer; (**c**) the lateral aspect of the ring transducer.

A ceramic plug (fiber-optic pins, RWD, R-FOC-BL200C-39NA, D = 1.25mm) was used to deliver the excitation light and collect the fluorescence signal. The ceramic plug was placed in the middle of the ring transducer, and the plug leaked out of the collimator’s end by 2 mm in order for the plug to reach a depth of 2 mm in the visual cortex. A fiber photometry system (RWD R810 Dual Color Multichannel) was used to emit the 470 nm excitation light and collect the fluorescence signal, selectively capturing the response from the visual cortex. The system setup can be seen in Figure 2.

#### 2.3.2. Electrophysiological Signal Acquisition

A collimator with a depth of 20 mm was used to ensure the positioning of the ultrasound focus. In the middle of the ring transducer, there are three tungsten microelectrodes (2 MΩ, Tungsten—Standard Tip—with Polyimide Tubing, MicroProbes, USA) and a reference electrode [36]. The head of the electrode is 3mm longer than the bottom end of the collimator. The electrode was aligned to the ultrasound focal area as closely as possible. The reference electrode was attached to the scalp, and the ground electrode was placed on the hindlimb. Signals were acquired by the headstage (Plexon, 20X, headstage), then amplified by a preamplifier (Plexon, 50X, bandpass filter 133 Hz–10 kHz), and then finally recorded by a PowerLab data acquisition (DAQ) system (Powerlab 8/30, ADInstruments, Sydney, Australia). The sampling frequency was 40 kHz. DAQ was synchronized with US and light stimulation.

### 2.4. Light and Ultrasound Stimulation and Signal Acquisition

Thirty days after the virus injection, the rats were anesthetized, and a 3 mm diameter hole was made above the visual cortex area. A 6-axis stereotaxic instrument was used to fix the ceramic pins, electrodes, and ultrasound probe. They were integrated together and slowly implanted into the primary visual cortex of the rats. The rats were then subjected to light stimulation and brain US.

In order to verify that the ultrasonic sound field does not cause slight vibrations in the electrodes and thus generate signal interference, an initial test was conducted. During this testing, the transducer was placed in water together with the electrodes, and the signal generator was set with the following parameters: drive voltage 33 V, center frequency 3.6 MHz, stimulation time 150 ms (cycle = 535 k), interval 20 s, and 50 repetitions.

For in vivo recordings in rats, a small window (2.5 mm × 2.5 mm) was opened above the VC for the insertion of the electrodes and ceramic pins approximately 1.2 mm below the skull using a 6-axis stage to reach the visual cortex.

To record light stimulation activities in the visual cortex, a full-field strobe flash using a Grass Photic stimulator (Grass Instrument Co., W. Warwick, RI, USA) was placed in front of the eye to deliver the flashlight stimulation. The optical stimulation had a duration shorter than <1 ms. 

During US, the signal generator was set up with the following parameters: drive voltage 33 V, center frequency 3.6 MHz, stimulation time 30 ms (cycle = 107 k) and 60 ms (cycle = 214 k), and interval 5 s. The electrophysiological signals and fiber-optic calcium imaging signals were acquired simultaneously.

## 3. Results

### 3.1. Acoustic Intensity Measurement

**Figure 3:**
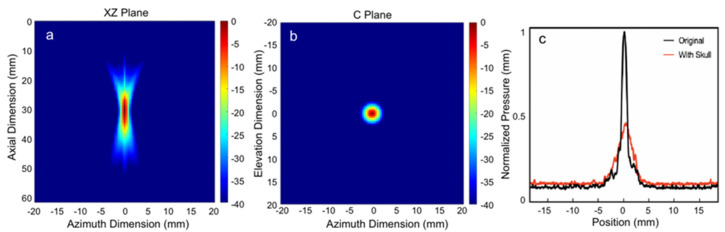
The results of the pressure distribution simulation performed using FIELD II. The pressure field was measured in two planes, the YZ Plane (**a**) and the C Plane (**b**), without the presence of the skull. To investigate the impact of the rat skull on the pressure distribution, 1D-scan measurements were performed using a hydrophone both with and without the skull (**c**).

The pressure distribution across various planes was simulated using FIELD II. In order to evaluate the influence of the skull on the pressure distribution in the rat model, hydrophone measurements were performed both with and without the cranium present. Figure 3a,b show the simulation results of the sound field power distribution of the 3.6 MHz annular transducer in the 2 mm × 2 mm axial plane (XZ plane) and transverse plane (C Plane), respectively. The lateral focal width at −3 dB is approximately 2 mm, and the lateral plane is the same as the axial plane.

In order to evaluate the impact of a rat skull on the sound field, a slice of the skull was positioned between the hydrophone and the transducer. Figure 3c illustrates the typical scenario and the results of the transcranial one-dimensional scan, with normalized pressure. The horizontal and vertical scan widths were 36 mm, with a distance of 72 µm between each scan step. Since it is a circular transducer, the horizontal width (YZ plane) should be identical to the XZ plane in the simulation. The peak-to-peak amplitude of the focused ultrasound pressure, as measured in the free field, was 3.6 MPa, and the mechanical index (MI) was 2.6. At a frequency of 0.5 MHz, the transmission factor of ultrasound through the adult rat skull was estimated to be 0.5~0.6, reducing the MI from 2.6 to 1.3~1.6 [7,37]. Since the age of each LE rat was the same, the influence of age was minimized. Therefore, the effect caused by the skull thickness of different rats did not affect the results of US stimulation and can be considered negligible. When the skull was positioned between the transducer and the hydrophone, a broadband attenuation of approximately 8 dB in the eight-annulus measurement was detected, which may have been due to additional defocusing effects from the larger surface exposed to the ultrasound. The results of the one-dimensional scan indicated that the attenuation caused by focused ultrasound through the skull was 51.5%. The average thickness of the skull, as determined through micro-CT scans, was 701 µm (SD = 65 µm), with the skull divided into front, middle, and back sections based on the Bregma and Lambda points. The mean thickness was 712 µm (SD = 59 µm) in the front, 694 µm (SD = 41 µm) in the middle, and 704 µm (SD = 55 µm) in the back. These measured values of bone attenuation were taken into account for the subsequent planning of therapy and in vivo experiments involving transcranial ultrasound stimulation.

### 3.2. Electrophysiological Results of Ring Transducer Stimulation of the Visual Cortex

**Figure 4:**
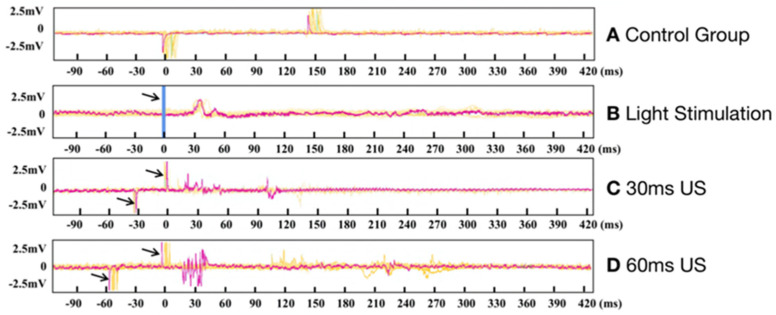
(**A**) The whole device placed in water (control experiment), (**B**) light stimulation (the arrows and blue line show the stimulation time), and there were some spikes after 30 ms, (**C**) 30 ms ultrasound stimulation on the visual cortex (the arrow indicates the position of the initial pulse), (**D**) the 60 ms ultrasound stimulus.

Figure 4 shows the results of non-invasive US on the rat VC without the removal of the skull. The control group data (Figure 4A, control experiment as described in the methodology) show that the electrode collected two pulses that were caused by electrical leakage of the ultrasound transducer. The yellow lines represent the overlay of eight sets of data, and the red lines represent one of them in this group. The amplifier connected to the transducer generated electromagnetic interference that was picked up by the electrode each time it was switched on and off; therefore, two pulses were collected by the electrodes, and the interception time corresponds to the stimulation duration. Between the two pulses, the signal waveform stayed relatively stable, demonstrating that the high-impedance electrode had a high signal-shielding capacity. US did not lead to electrode vibration, and thus, the neural signal was not disturbed. Notably, the neuronal spike evoked by light stimulation occurred within 30 ms after the end of light stimulation, and the neuronal response evoked by US (30 ms duration) occurred in the VC within 15 ms to 120 ms after stimulation. The response speed of the VC neurons elicited by US is faster compared to that elicited by light stimulation. Longer US (60 ms duration) evoked a neuronal response in the VC, within 10 ms to 300 ms after US. Light stimulation of the retina took more time to evoke a neuronal response compared to the direct stimulation of the visual cortex; this phenomenon could be due to the fact that light stimulation has to travel through the optic nerve.

### 3.3. Photometry Results of Ring Transducer Stimulation of the Visual Cortex

**Figure 5:**
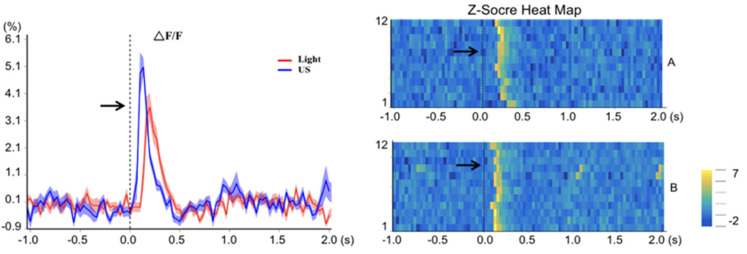
Results of ultrasound-stimulated fiber-optic calcium imaging of rat visual cortex. The red line represents the change in ΔF/F for light stimulation, and the blue line represents ultrasound stimulation. (**A**) The 12-times stimulation results of ΔF/F for flashlight stimulation; (**B**) the 12-times stimulation results of ΔF/F for US stimulation.

The photometry results can be seen in Figure 5, which illustrates the neuronal response evoked by both light stimulation and US. The pre-trigger signal indicated by the arrow represents the actual US given. A Z-Score heatmap was calculated based on the normalized data counts across 12 trials in each group. Notably, US (30 ms) evoked a faster neuronal response in the VC than light stimulation, which also corresponded to the EEG signal shown in Figure 4 for demonstrating a more rapid response to US.

## 4. Conclusions and Discussion

In this experiment, we utilized US to target the VC and recorded the response of neuronal activities in the VC using electrophysiological and fiber-optic calcium signals in a rat model. Our results showed that the presence of the skull decreased the MI from 2.6 to 1.3~1.6 due to the thickness of the skull. This suggested that the ultrasound sound field could only partially penetrate the skull. Skull-penetrating ultrasound can still elicit a meaningful neuronal response, as indicated by changes in the electrophysiological and calcium signals.

One of the key issues to be addressed in non-invasive deep brain neuromodulation using tFUS is how to overcome the effects of the skull on ultrasound. The density of and speed of sound in the skull are approximately twice those of other human soft tissues, and the sound attenuation coefficient is at least an order of magnitude higher. Furthermore, the skull has a complex non-homogeneous structure with multiple layers and fluid-filled and porous features, causing significant phase distortion and energy attenuation when ultrasound passes through it. However, the use of ultrasound phased arrays can help overcome the obstacles caused by the skull in tFUS: 1. Using magnetic resonance imaging (MRI) and MRI-compatible ultrasound phased arrays can provide an imaging guide for the treatment process of brain diseases with focused ultrasound. 2. To compensate for the effect of attenuation and aberration from the skull, an MRI or CT scan of the skull can be used to establish three or even more layers of non-uniform skull density and speed-of-sound models. Then, theoretical modeling and numerical simulations can be used to calculate the phase compensation required by the ultrasound phased array in tFUS to achieve a better transcranial focus effect [38,39]. Non-invasive tFUS technology that does not require a craniotomy is becoming increasingly possible and attracting more and more attention and research.

The duration of the excitation time of VC neurons increased with a longer duration of US. Moreover, we observed that the response speed of VC neurons elicited by US is faster than that elicited by light stimulation, which may be a result of the time required to excite retinal photoreceptor cells, followed by signal transfer to the VC. This low-latency advantage of the US VC prosthesis can be used to compensate for the internal computational time delay of the system caused by the head-mounted camera image acquisition system converting the data to 2D patterning for stimulating the visual cortex, helping to develop faster system response solutions. The fiber photometry data confirmed that non-invasive US was capable of eliciting a response in the VC, and its response speed was also faster than that of light stimulation.

It is possible that ultrasound may cause bone conduction and elicit a response in the auditory cortex, which is close to the visual cortex. As a result, the electrophysiological signals may be disturbed by the auditory cortex [40,41,42]. Some researchers [41] have used chemical or physical methods to induce deafness in rats stimulated by ultrasound in order to avoid the influence of auditory cortex responses. In this experiment, since the injection of the GCaMP8m virus was limited to the visual cortex, calcium imaging showed that the induced responses were only from the visual cortex. Furthermore, we observed from EEG signals and calcium imaging signals that the neuronal response time induced by ultrasound was faster than that induced by light stimulation, while inducing auditory cortex responses to further transmit to the visual cortex required a longer time delay, thereby further excluding the influence of the auditory cortex. In future research, we plan to inject the virus into the auditory cortex and, when testing the response of the visual cortex to ultrasound, use calcium imaging to describe any interference from the auditory cortex.

The physical mechanism of in vivo ultrasound neuromodulation is another important yet undetermined issue. Candidates for the physical mechanisms of ultrasound stimulation include cavitation, acoustic radiation force (ARF), acoustic streaming, and thermal effects. First, it is generally accepted that the heating effect of US is minimal when the stimulation duration is brief (in the order of milliseconds) and the pulse repetition rate (PRF) is low [43,44]. Other US neuromodulation studies have also used parameters that support the notion that temperature increases are not a significant concern in well-controlled US. Li’s [28] work demonstrated that the maximum temperature rise of optically generated focused ultrasound (OFUS) was assessed to be <0.1 K. This is far lower than the threshold required for the thermal regulation of neuronal activity (ΔT ≥ 5 K). Furthermore, the thermal effect is a gradually accumulating effect that deposits energy in brain tissue and has a longer latency response time. Since our ultrasound stimulation and light stimulation have almost the same response time, the thermal response can be ruled out. Cavitation refers to the phenomenon that occurs when ultrasound waves interact with a liquid. When the acoustic pressure reaches a sufficiently negative peak, vapor bubbles can be created as a result. The size of the bubbles oscillates with the sinusoidal change in local pressure. Instantaneous cavitation occurs when the bubble size increases at least twofold [45]. In stable cavitation, the size changes are smaller, and the bubble does not rupture, which is speculated to produce stable neuromodulation [46]. Acoustic streaming is the linear mechanical effect of sound waves, causing the tissue to oscillate sinusoidally with a much lower amplitude compared to cavitation. ARF is a nonlinear acoustic effect that generates a non-oscillatory force, causing the unidirectional displacement of biological tissue [47]. It is speculated that all mechanical acoustic effects would influence local neural ion channels, promoting cellular ion fluxes and inducing neuronal electrical activity [48].

Based on the experimental results in this paper, since the MI is the index for cavitation and the MI in our experiment is lower than the FDA-required 1.9, the cavitation effect should not be a significant factor. We tend to emphasize the key role of ARF. ARF has a non-oscillatory unidirectional effect, while stable cavitation and acoustic streaming are sinusoidal and periodic effects. Ultrasound can push neurons or ion channels, causing measurable potentials, where both excitatory and inhibitory neurons are stimulated, and the accumulated action potentials are collected by the electrodes, forming the current non-periodic signal.

Due to limitations in terms of stimulation safety and the effects of ultrasound heating, the strength of the ultrasound field cannot be increased indefinitely. In the future, a mediator s MscL-G22s may be used to enhance neuronal response sensitivity. MscL-G22s is a highly conductive mechanically sensitive ion channel family composed of pore-forming membrane proteins that can convert physical forces applied to cell membranes into electrophysiological activity. It has been proven that only neurons in the brain region that have been injected and express MscL-G22s are activated by ultrasonic stimulation, while neurons in other regions that have not been injected with MscL-G22s do not undergo significant activation [49]. As a result, the ultrasound genetics of MscL-G22s is a potentially promising tool for restoring vision and treating other brain diseases.

Focused ultrasound with a center frequency higher than 3 MHz has the potential to stimulate the visual cortex with a spatial resolution finer than 0.5 mm, which is comparable to the size of the most advanced electrodes used in Visual Cortical Prosthesis (VCP). This study may benefit from using a 2D ultrasound array instead of a single-element transducer, as it allows for electrical steering and the generation of arbitrary 2D patterns by adjusting the phase and amplitude of each element in the array. Camera-acquired images will be used to control stimulation by converting them into ultrasound patterns. The amplitude and phase distributions of the 2D array will be calculated using ultrasonic backpropagation algorithms [50,51,52]. This research will be a necessary step in realizing a non-invasive and effective VCP.

Based on the discussion above, visual cortical prostheses may provide an intervention point for almost all forms of blindness, including those caused by optic nerve disease and damage to the prefrontal cortical area. Our proposed non-invasive, ultrasound-based VC prosthesis has the potential to eliminate many of the complications associated with electrode-based visual cortical prostheses. With further development, US technology may provide a non-invasive method of visual restoration for blind patients with optic nerve pathology.

## Data Availability

The data presented in this study are available on request from the corresponding author.
